# Endotoxemia in pediatric critical illness - a pilot study

**DOI:** 10.1186/cc10264

**Published:** 2011-06-08

**Authors:** Shamik Dholakia, David Inwald, Helen Betts, Simon Nadel

**Affiliations:** 1 Department of Paediatric Intensive Care, St Mary's Hospital, Imperial College Healthcare NHS Trust and Imperial College London, Praed Street, London, W21NY, UK; 2 Department of Paediatrics, Imperial College London, Norfolk Place, London, W22PG, UK

## Abstract

**Introduction:**

The aim was to investigate the prevalence of endotoxemia in children admitted to pediatric intensive care unit (PICU), and its association with disease severity and outcome.

**Methods:**

We conducted a prospective, observational cohort study of children admitted to PICU at St. Mary's Hospital, London over a 6-month period. One hundred consecutive patients were recruited. Demographic and clinical data were collected. Severity of illness was assessed by the pediatric index of mortality 2 (PIM2) score. The pediatric logistic organ dysfunction (PELOD) score was performed daily for the first 4 days. Patients were categorized according to primary reason for PICU admission. Blood samples were taken within 24 hours of admission and endotoxemia was measured using the endotoxin activity assay (EAA). Patients were stratified according to EAA level (high, EAA > 0.4, low, EAA < 0.4) and categorized as septic, post-surgical, respiratory or other. Data were analyzed using appropriate non-parametric tests.

**Results:**

EAA level was significantly lower in PICU controls versus other PICU admissions (*P *= 0.01). Fifty-five children had endotoxemia on admission. Forty-one (75%) of these were eventually diagnosed with an infectious cause of admission. Nine children without infection had elevated EAA on admission. An infectious cause of admission was significantly associated with endotoxemia (*P *< 0.005). Of 15 children with gram-negative infection, only 9 (60%) had endotoxemia on admission. Endotoxemia on admission was not associated with shock or death. However, there was a tendency for increased PELOD score and length of stay in endotoxemic children.

**Conclusions:**

Endotoxemia is common in children admitted to intensive care. Understanding the implications of endotoxemia and potential anti-endotoxin strategies may have the potential to reduce severity of illness and length of PICU stay in critically ill children.

## Introduction

Sepsis is a major cause of admission to pediatric intensive care units (PICUs) and causes significant morbidity and mortality in children. A recent study from the US estimated that the incidence of pediatric severe sepsis is 0.56 cases per 1,000 population and that severe sepsis has an overall hospital mortality rate of 10.3% and accounts for 7% of all deaths in children [1].

In 2009, there were nearly 17,000 admissions to PICUs in the UK. Of these, nearly 9,000 (53%) were unplanned medical admissions for conditions such as sepsis, pneumonia, bronchiolitis, and respiratory failure (Pediatric Intensive Care Audit Network [PICANET] Annual Report 2009) [2]. The unadjusted case fatality rate for children admitted to PICUs in the UK is 4.1% and these children account for over 100,000 bed days. A recent audit of referrals of children with sepsis to PICUs in the UK found that 17% of these children died [3].

The earliest events in the pathogenesis of sepsis are interactions of pathogen-related antigens (for example, endotoxin (lipopolysaccharide, LPS) and peptidoglycan) with cell surface pattern recognition receptors such as the Toll-like receptors 4 and 2, respectively [4]. The interaction of LPS with its receptors and the subsequent cellular responses have been well described and are pivotal processes in the inflammatory response leading to the manifestations of sepsis [5].

Measuring the concentration of LPS in human disease has been notoriously difficult. The most commonly used method, the chromogenic limulus amebocyte lysate (LAL) assay, is based on the ability of endotoxin to induce coagulation of hemolymph in the horseshoe crab, *Limulus polyphemus *[6]. The utility of this assay has been limited because of circulating inhibitors of the coagulation reaction. In addition, the assay is not specific for endotoxin.

A novel endotoxin assay, which is simpler and more accurate than the LAL assay, was recently described [7]. This endotoxin activity assay (EAA) detects LPS in whole blood by the use of neutrophil-dependent chemiluminescence. This assay was used in a study of critically ill adults, in which an association between endotoxemia with infection and an increased risk of adverse outcome was demonstrated [8]. Because of the link between endotoxin and inflammation, we sought to define the prevalence of endotoxemia in critically ill children and determine the association of endotoxemia with infection, severity of illness, and outcome.

## Materials and methods

We undertook a prospective observational cohort study of critically ill children admitted to the PICU at St Mary's Hospital, London, over the course of a 6-month period (January to June 2007). The unit does not admit cardiac-surgical or neurosurgical patients. Informed consent was obtained from parents or carers. The study was approved by the local research ethics committee.

Demographic and clinical data were collected on admission to the PICU. Patients were categorized by their primary reason for admission: respiratory failure (that is, acute viral bronchiolitis, croup, asthma, or pneumonia), neurological failure (that is, meningo-encephailitis or status epilepticus), sepsis, cardiac (that is, myocarditis or arrhythmia), surgical, or other (that is, trauma or cardiac arrest).

We defined a PICU control group as children who were electively admitted to the PICU for post-operative care. They were all electively ventilated but had no other organ failure on admission.

Conditions were diagnosed as sepsis or septic shock (or both) according to the criteria of Goldstein and colleagues [9]. Infection was diagnosed with standard microbiological techniques.

Severity of illness was assessed by the pediatric index of mortality 2 (PIM2) score [10], and the pediatric logistic organ dysfunction (PELOD) score [11] was performed daily for the first 4 days (as 4 days was the mean length of stay, or LOS).

### Chemiluminescent assay for endotoxin

All patients had a single measurement of endotoxin activity (EA) in whole blood within 24 hours of PICU admission, as described previously [7]. Whole blood samples (2 mL) were collected into ethylenediaminetetraacetic acid (EDTA) vacutainer tubes. Samples of 0.5 mL of whole blood in duplicate were immediately incubated with saturating concentrations of a murine IgM monoclonal antibody against the lipid A of *Escherichia coli *J5. This antibody is broadly cross-reactive against Gram-negative bacteria but does not cross-react with Gram-positive bacteria. Any LPS present in the blood complexes with the anti-LPS antibody. This complex primes the patient's neutrophils for an augmented response to stimulation with zymosan. The resulting respiratory burst activity is detected as light release from the lumiphor luminol by using a chemiluminometer (Autolumat LB953; EG&G Berthold, Bad Wildbad/Germany). By measuring basal (no antibody) and maximally stimulated (4,600 pg/mL LPS) responses in the same blood sample, the EA of the test specimen is calculated by integrating chemiluminescence over time. Levels are expressed as EA units and represent the mean of duplicate determinations from the same sample. An EA level of greater than 0.4 is approximately equivalent to an endotoxin concentration of 25 to 50 pg/mL *E. coli *055:B5 LPS.

As the EAA requires adequate numbers of patient neutrophils to provide the respiratory burst for the assay readout, we excluded patients who had an absolute neutropenia of less than 0.1 × 10^6^/cm^3^. For the purposes of this study, a cutoff level of 0.4 EA units, as defined previously [7], was used to determine the presence of endotoxemia (that is, ≤0.4 negative, > 0.4 EA present).

### Statistics

Data were non-parametric and are presented as median ± interquartile range (IQR). The results were analyzed by using GraphPad Prism (GraphPad Software, Inc., La Jolla, CA, USA). The Mann-Whitney *U *test was used to compare groups of data, and the Kruskal-Wallis test was used if there were more than two groups of data. The chi-square test was used to compare proportions.

## Results

One hundred four consecutive admissions were asked to participate in the study but four of them refused. No children were excluded; therefore, 100 patients were recruited for the study. Their demographics are shown in Table 1. Ten children (10%) died and 90 were discharged alive from the PICU. The mean LOS for survivors was 7.1 days (median of 5 days and range of 1 to 57). There were 9 children in the control group, 48 in the respiratory group, 18 in the sepsis group, and 25 in the 'other' group. The control group consisted of two children who had tonsillectomy and adenoidectomy for obstructive sleep apnea, three children who had a Nissen fundoplication and gastrostomy, one child who had a gastric pull-up for esophageal atresia, one child who had an esophageal dilatation, one child who had a resection of a colonic duplication cyst, and one child who had a partial nephrectomy. None suffered periods of intra-operative hypotension or needed prolonged post-operative ventilation.

**Table 1:** Demographic data

	Number
Total population recruited	100
Age in months	
Median	33.8
Range	0-180
Males	63
Diagnosis	
Respiratory^a^	48
Sepsis	18
Other^b^	25
Control^c^	9
Shock on admission	34
Died before discharge	10

^a^Includes eight patients with respiratory syncytial virus bronchiolitis. ^b^Includes seven patients with primary neurological failure and four patients with primary cardiac failure. ^c^Post-operative elective admission. Patients may fall into more than one category.

**Figure 1:**
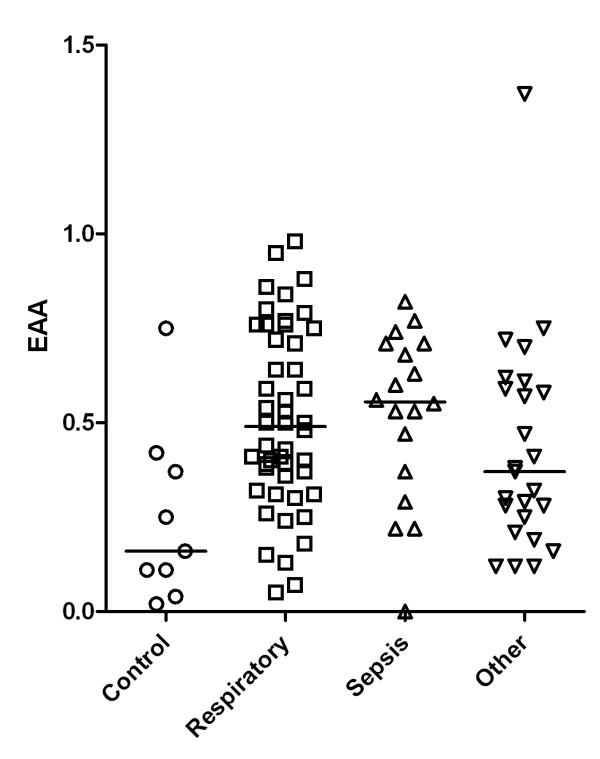
**Endotoxin activity assay (EAA) level related to clinical classification**. For a statistical analysis, see the 'Statistics' section.

EAA level was significantly lower in the control group (0.16, IQR 0.11 to 0.37) compared with the respiratory (0.48, IQR 0.34 to 0.74), sepsis (0.55, IQR 0.4 to 0.7), and other (0.37, IQR 0.25 to 0.59) groups (*P *= 0.01, Kruskal-Wallis test) (Figure 1).

Fifty-five children had endotoxemia on admission to the PICU. Only two of these were in the control group (one child underwent an elective Nissen fundoplication and had extensive bowel manipulation during surgery, and the other child underwent an adenotonsillectomy). Of these 55 children, 41 (75%) were eventually given a diagnosis of an infection-related cause of admission (Table 2).

**Table 2:** Infectious causes of admission (*n *= 62)

	Number
Viral	
Adenovirus	3
Influenza A	2
HHV6	1
Metapneumovirus	2
Parainfluenza 1	1
RSV	8
Varicella	2
Bacterial	
*Staphylococcus aureus*	8
Group A Streptococcus	4
Group B Streptococcus	2
*Haemophilus influenzae*	3
Klebsiella spp.	1
Meningococcus	6
Pneumococcus	8
Pseudomonas	4
Salmonella spp.	1
Other	
PCP	2
Malaria	1
Aspergillus	1
Candida	2

HHV6, human herpes virus 6; PCP, pneumocystis jirovecii pneumonia; RSV, respiratory syncytial virus.

Seventeen children were bacteremic on admission. Fifteen children had Gram-negative infection as the cause of admission, and only nine (60%) of them were endotoxemic on admission. Of the 15 children with Gram-negative infection, only 5 had Gram-negative bacteremia (all meningococcus serogroup B). Only 3 of the 5 children with meningococcal bacteremia had endotoxemia detected on admission.

Sixty-two children were given a diagnosis of an infection-related cause of admission, and 41 (66%) of them had endotoxemia and 14 of 38 children (37%) had a non-infectious cause of admission (*P *= 0.0043, chi-square test). Seventy-five percent of children with respiratory failure of any cause had endotoxemia on admission to the PICU.

All children admitted to our PICU with a suspicion of infection are treated with a standard regimen of anti-microbials, consisting of a third-generation cephalosporin (usually ceftriaxone) with or without gentamicin if there is a suspicion of pseudomonas infection (such as patients with chronic lung disease). There was no clear correlation between anti-microbial use and endotoxemia (data not shown).

The presence of endotoxemia on admission to the PICU was not significantly associated with shock or death. Twenty-two of 34 children (64%) who had shock at PICU admission had endotoxemia compared with 35 out of 66 (53%) without shock (*P *= not significant, chi-square test). Seven of the 10 children (70%) who died had endotoxemia on admission compared with 48 out of 90 survivors (53%) (*P *= not significant, chi-square test). Daily PELOD score, PIM2 score on admission, and LOS in survivors were not significantly different between the groups with and without endotoxemia on admission, although there was a tendency for increased PELOD score and LOS in the group with endotoxemia (Table 3).

**Table 3:** Severity of illness

	EAA ≤0.4	EAA > 0.4	Mann-Whitney
PIM score, median (IQR)	5% (1.5%-13%)	6% (2%-20%)	*P *= 0.35
PELOD score, median (IQR)	1.7 (1.15-20.8)	16.2 (1.3-20.8)	*P *= 0.31
LOS of survivors, median (IQR)	4 (2-7)	5 (4-8)	*P *= 0.05

EAA, endotoxin activity assay; IQR, interquartile range; LOS, length of stay (days); PELOD, pediatric logistic organ dysfunction; PIM, pediatric index of mortality.

## Discussion

Our study shows that endotoxemia is common in a heterogeneous population of critically ill children. Although there was no clear correlation with severity of illness or the presence of shock, there was a tendency toward longer LOS in patients with endotoxemia. This is consistent with a recent study in adult surgical intensive care patients, which showed an association between endotoxemia and LOS in the intensive care unit [12].

Despite the high prevalence of endotoxemia found in our study, only 15 children had a Gram-negative infection, a scenario classically associated with endotoxemia. In addition, only 9 of 15 children with documented Gram-negative infection had endotoxemia. This may reflect the timing of the assay in relation to admission and treatment. It has previously been noted that endotoxin level in blood declines rapidly following appropriate anti-microbial therapy [13]. We did not record the timing of performance of the EAA in relation to the start of anti-microbial therapy, but 75% of the samples were taken within 12 hours of admission to the PICU.

The commonest cause of PICU admission in our study was respiratory failure that was usually due to viral lower respiratory tract infection. The majority (75%) of children with a respiratory tract infection had endotoxemia on admission despite having low severity-of-illness scores and good outcome. Several studies have documented high levels of endotoxin in the lung and blood of patients with respiratory infection as well as those on artificial ventilation. Pulmonary-to-systemic translocation of LPS has been demonstrated in non-protective ventilation strategy studies in experimental animals [14], and an association between ventilator-induced lung injury, ventilator-associated pneumonia, and systemic LPS and cytokine levels has been reported [15]. Although the lung has no endogenous microflora that could provide a source of LPS, it is likely that bacterial colonization of the airway or lung may be a potential source of LPS that can translocate systemically following mechanical ventilation. The high prevalence of endotoxemia in our patients seems to confirm this.

The gut provides a second and major potential source of systemic LPS. The indigenous flora of the gastrointestinal tract contains large amounts of endotoxin, and translocation of both endotoxin and viable bacteria from the gut has been demonstrated in animal models and in human illness associated with splanchnic hypoperfusion [16,17]. Consistent with this, we observed that endotoxemia was more common in critical illness compared with control children. In addition, patients who had endotoxemia on admission appeared to be a sicker population, as reflected by longer LOS and a trend toward higher PELOD scores. Thus, the presence of endotoxemia on admission may identify a high-risk subpopulation of critically ill children.

The chemiluminescent assay used in our study is both sensitive and specific for endotoxin and can be performed in less than 1 hour, permitting the rapid detection of endotoxin in fresh whole blood. However, it uses the patient's own neutrophils as a readout system and so presents inherent limitations; in particular, it is not possible to store specimens for later assay; measurements must be performed within 3 hours of obtaining the sample. Because of the requirement for neutrophils, the assay is not reliable in patients with an absolute neutrophil count of less than 0.1 × 10^6^/cm^3^. The assay relies on neutrophil chemiluminescence in response to IgM anti-LPS antibody and LPS. However, the pre-activation state is accounted for by correcting for baseline chemiluminescence in the EAA. In addition, the assay detects exposed lipid A in the endotoxin molecule and so may not reflect endotoxin bioactivity *in vivo*. However, the availability of a relatively simple bedside assay for blood endotoxin level may identify a high-risk population of critically ill patients who may benefit from adjunctive therapy.

We did not correlate the EAA with the best-known endotoxin assay, the LAL assay. The LAL assay has been widely used to detect endotoxin contamination of drugs and fluids; however, its utility in biological samples has been limited because of circulating inhibitors of the coagulation reaction. In addition, other microbial products, notably from fungi, can activate the LAL reaction, so the assay is not specific for endotoxin [18]. Studies comparing EAA and LAL show considerable variability in the prevalence of endotoxemia or its association with Gram-negative infection, and the EAA is able to detect endotoxemia associated with Gram-negative infection from any source, a diagnosis of sepsis, and an elevated white blood cell count; no such correlations were found when samples were assayed using the LAL method [19].

Antibiotics can accelerate endotoxin release and may result in false-negative blood cultures [20]. We did not correlate endotoxemia with type and duration of anti-microbial therapy. However, most patients would have been treated with our typical empiric anti-microbial regimen, which consisted of a third-generation cephalosporin, with additional gentamicin in certain cases, such as those with specific risk factors for pseudomonas infection.

Whether the increased LOS and possible increased severity of illness associated with endotoxemia might be reduced by specific measures to neutralize endotoxin needs to be studied further, but the hypothesis remains attractive. High levels of endotoxin have been associated with increased severity of illness in meningococcal disease [13], and studies of anti-endotoxin therapy in human sepsis suggest that the greatest potential benefit occurs in patients in whom endotoxemia is likely to be present [21].

Inferences from the present study are limited by its observational nature and the relatively small number of patients in each disease category. In addition, the timing of the assay may have 'missed' the peak of endotoxemia in those patients in whom anti-microbial therapy had been initiated prior to PICU admission. In addition, intrinsic anti-EA (such as endotoxin antibodies) and lipoproteins may quench EA.

## Conclusions

The demonstration of significant endotoxemia in a high proportion of children admitted to the PICU for any one of several pathologies suggests an important interaction between critical illness and inflammation induced or augmented by intrinsically derived endotoxin. It is possible that this endotoxemia contributes to severity of illness and outcome. The hypothesis that LPS contributes to disease severity in critical illness requires further exploration. Our study suggests that endotoxemia in critically ill children is common and can be detected with a simple bedside test. The use of anti-endotoxin agents potentially may have a role in the treatment of critical illness of all causes in children, and this should be the subject of future studies.

## Key messages

• Children with critical illness of any cause are likely to have endotoxemia.

• Endotoxemia can be accurately detected by a simple bedside test.

• Endotoxemia may be associated with increased severity of illness and length of stay in the pediatric intensive care unit.

• It is likely that the source of endotoxin in these children is their own gut or lung.

• If the findings of this study are confirmed in larger studies, anti-endotoxin therapies may be a logical adjunct to the treatment of pediatric critical illness.

## Abbreviations

EA, endotoxin activity; EAA, endotoxin activity assay; IQR, interquartile range; LAL, limulus amebocyte lysate; LOS, length of stay; LPS, lipopolysaccharide; PELOD, pediatric logistic organ dysfunction; PICU, pediatric intensive care unit; PIM2, pediatric index of mortality 2.

## Competing interests

The authors declare that they have no competing interests.

## Authors' contributions

SD recruited patients, performed the assay, and wrote the first draft of the manuscript. DI gave statistical advice and contributed to the manuscript. HB recruited patients, performed the assay, and contributed to the manuscript. SN initiated the project, recruited patients, performed the assay, and contributed to the manuscript. All authors read and approved the final manuscript.
